# Ventromedial prefrontal cortex mediates sex differences in persistent cognitive drive for food

**DOI:** 10.1038/s41598-018-20553-4

**Published:** 2018-02-02

**Authors:** Lauren C. Anderson, Gorica D. Petrovich

**Affiliations:** 1 Department of Psychology, Boston College, Chestnut Hill, MA USA; 2 Department of Neurology, Harvard Medical School/Massachusetts General Hospital, Charlestown, MA USA

## Abstract

Contemporary environments are saturated with food cues that stimulate appetites in the absence of hunger, which leads to maladaptive eating. These settings can induce persistent drive to eat, as learned behaviors can reappear after extinction. Behavioral paradigms of responding renewal provide a valuable framework to study how food cues contribute to the inability to resist palatable foods and change maladaptive eating habits. Using a rat model for this persistent food motivation, we determined sex differences in the causal function for the ventromedial prefrontal cortex (vmPFC) during context-mediated renewal of responding to food cues. Previously, we found behavioral sex differences (only males exhibited renewal) and differential recruitment within the vmPFC (increased Fos induction in males but decreased in females). Here, we used DREADDs (Designer Receptors Exclusively Activated by Designer Drugs) to silence vmPFC neurons in males and to stimulate vmPFC neurons in females specifically during renewal. Silencing vmPFC neurons in males disrupted renewal of responding to a food cue, while stimulating vmPFC neurons in females induced this behavior. These findings demonstrate sex differences in the vmPFC function in a model of food seeking relevant to environmentally driven appetites contributing to obesity and eating disorders.

## Introduction

Persistent food cravings and the inability to resist palatable foods even when eating is maladaptive are hallmarks of overeating dysregulation^1^. Food cues are important contributors to these excessive drives. Cues previously associated with food can stimulate appetite and food consumption in the absence of hunger, which can lead to maladaptive overeating^2–5^. Food cue exposure, and associated cravings, significantly influence eating behavior and weight gain in humans^6^ and neural responding to food cues in sated states has been correlated with long-term weight gain^7^. A sharp rise in obesity has occurred over the last 30 years, and during the same period there have been profound changes in the locations where we consume food. The number of meals consumed outside the home has substantially increased, and these meals occur in distinct places such as fast-food restaurants^8^. Palatable foods are now so widely available that very few locations in our environments are not associated with food and eating.

Environments (contexts) previously associated with food can later stimulate consumption in the absence of hunger^9^. In addition to this function as conditioned stimuli, contextual cues are critical mediators of food memory and reappearance of learned behaviors after extinction. Cognitive processes mediating learned behaviors after extinction, such as renewal, might explain the difficulty associated with changing unhealthy eating habits and weight control in our environment^1,10^. Indeed, the renewal (reinstatement) model was recently introduced as a framework to study mechanisms of relapse to palatable food seeking, similar to the reinstatement model for relapse of drug use^1,11,12^.

One important renewal mechanism is mediated by context. Contexts can mediate memory formation and later recall of discrete cues (e.g., a tone or light), particularly when those cues have different meaning in different contexts. When a cue is paired with food in one context but not in another, context is ‘setting the occasion’ for the cue’s meaning—signaling food or not signaling food^13^. In that preparation, acquisition of a cue-food association and extinction of that association occur in different contexts, and then the renewal of conditioned behavior (food seeking) is induced by return to the acquisition context. Context-mediated renewal is a well-suited behavioral model for persistent food motivation, relevant to our contemporary lifestyle and insatiable appetites.

Women are more likely than men to be overweight and obese, and obese women show greater impairments in food associative learning^14–17^. There are also sex differences in animal models of associative learning, particularly with contextual processing^18–23^, that underscore the importance of determining mechanisms in both sexes. Still, female subjects are underrepresented in both basic and clinical research^24,25^. Therefore, recently we began to investigate both sexes in context-mediated renewal of responding. We found sex differences in context-mediated renewal of responding to Pavlovian food cues^19^, and distinct recruitment of the ventromedial prefrontal cortex (vmPFC) in male and female rats^20^. Increased Fos induction within the infralimibic and prelimbic areas in the vmPFC were associated with renewal behavior in males, while females did not show renewal or an increase in Fos induction. These distinct patterns suggest sex-specific vmPFC activation mediates behavioral sex differences. We posit context-mediated renewal is a decision-making process that involves retrieval, evaluation, and the use of context-mediated cue memory to guide responding, a function that requires vmPFC activation.

Here we aimed to establish the necessity of the vmPFC and that its differential activation underlies sex differences in context-mediated renewal of responding to Pavlovian food cues. First, we established an ABA renewal procedure that is suitable for within-subject neural manipulations. To directly test the causal function of the vmPFC we used a chemogenetic approach to manipulate vmPFC neurons to disrupt renewal of responding in males and to selectively induce this behavior in females. We used DREADDs (Designer Receptors Exclusively Activated by Designer Drugs)^26^ to silence (hM4Di) vmPFC neurons in males and activate (hM3Dq) vmPFC neurons in females during renewal tests. Two control conditions were employed in order to establish specific DREADDs effects. One group of control rats received DREADDs but was given a vehicle at test. Rats in the other control group received a control viral vector without DREADDs (AAV-only) and were given CNO at test. This CNO group was added in order to rule out potential effects of CNO independent from its action on DREADDs. Inclusion of that control group was particularly important given recent evidence that CNO was not biologically inert in some cases^27,28^. Identifying the vmPFC as a critical site of sex differences in context-mediated renewal will further our fundamental understanding of contextual processing and learning and memory in both sexes, as well as clinical treatment of excessive cognitive drive to eat.

## Results

### Experiment 1

**Figure 1:**
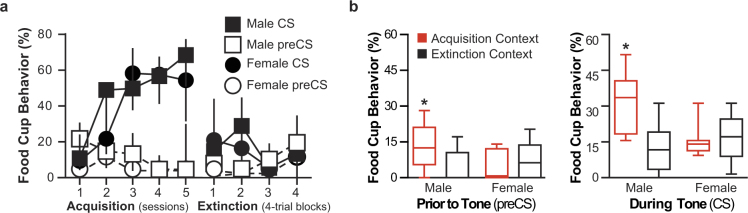
Conditioned responses to a food cue (CS) during acquisition, extinction and tests for renewal in Experiment 1. (**a**) Percentage of time rats expressed food cup behavior (median, error bars = inner quartiles) during the preCS (open) and CS (filled) periods across training sessions. Acquisition is shown as the average responding during each session. Extinction is shown as the average responding in 4-trial blocks (2 blocks per session; blocks 1&2 were trials during Session 1 and blocks 3&4 during Session 2). Acquisition and extinction training occurred in different contexts. Squares represent males; circles represent females. (**b**) Percentage of time rats expressed food cup behavior (box plots showing min, inner quartiles, median, and max) during the period prior to the tone (preCS) and during the tone (CS) in the Acquisition (shown in red) and Extinction Contexts (shown in black). n = 8 for both males and female groups.

In order to manipulate vmPFC neurons in a within-subjects preparation, we first established a behavioral procedure (Methods, Fig. 1a,b, Supplementary Fig. 1). All rats received acquisition training in a distinct context, consisting of tone (conditioned stimulus; CS) and food (unconditioned stimulus; US) pairings and then extinction training, consisting of CS presentations alone in a different context. Rats were then tested for food seeking conditioned responding (CR; food cup behavior) during CSs in each context. Higher responding during the CS in the acquisition context (vs. the extinction context) indicates successful renewal. We replicated the sex differences found with a between-subjects ABA paradigm^19,20^. Both sexes showed similar acquisition learning and similar performance during extinction training (Fig. 1a). At test, males showed significantly higher responding in the acquisition context compared to the extinction context during the CS, while females showed similar low responding in both contexts (Fig. 1b). A mixed-design ANOVA (repeated (context) and between (sex)) found a significant effect for Context by Sex interaction (F(1,14) = 19.483, *p* = 0.001). *Post hoc* two-tailed *t*-test determined males showed significantly higher responding in the Acquisition context (*p* < 0.05; Fig. 1b). Males also responded significantly higher during preCS in the acquisition compared to extinction context, while females had low responding in both contexts (mixed-design ANOVA (repeated (context) and between (sex)) significant for Test Context by Sex interaction (F(1,14) = 10.286, *p* = 0.006); *post hoc* two-tailed *t*-test (*p* < 0.05); Fig. 1b). Additional two-tailed t-test determined males showed significantly higher responding during the CS compared to preCS period in Acquisition context (*t*(7) = −4.257, p = 0.004). The elevation (CS-preCS responding) difference in responding in the acquisition vs. extinction context was also higher for males compared to females but that difference was not statistically significant (Supplementary Fig. 1).

### Experiment 2

**Figure 2:**
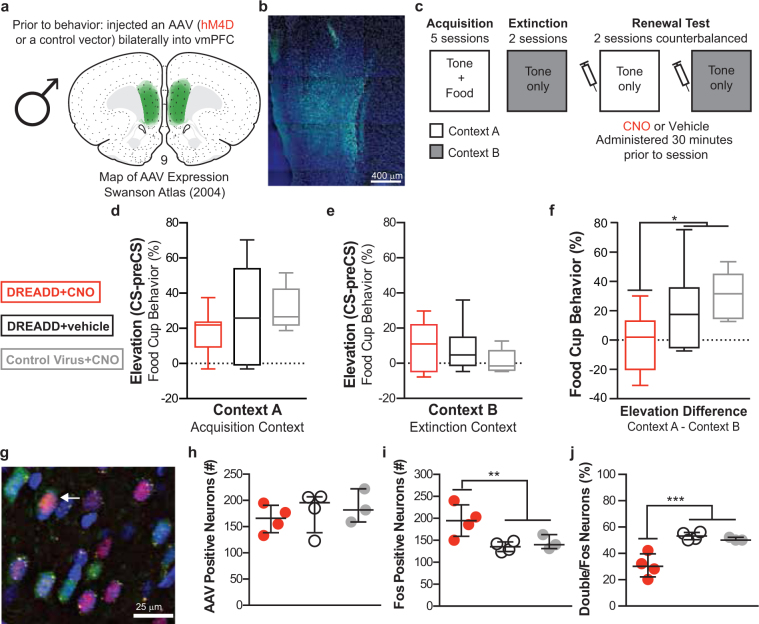
Chemogenetic vmPFC silencing in male rats during renewal of conditioned responding to a food cue. The extent of AAV expression within the vmPFC is shown on a modified atlas template (Swanson 2004) (**a**) and a representative image (**b**). Experimental design (**c**). Conditioned responding in the acquisition context (**d**) and the extinction context (**e**) and as a difference in responding between the two contexts (**f**), Representative image of AAV-positive (green), Fos-positive (magenta), and double-labeled (arrow) neurons (**g**). Total number of AAV-positive (**h**) or Fos-positive (**i**) neurons. Percentage of Fos-positive neurons that were double labeled (**j**). DREADD + CNO group (n = 7 behavior, n = 4 Fos) in red, DREADD + vehicle (n = 6 behavior, n = 4 Fos) in black, Control Virus + CNO (n = 5 behavior, n = 3 Fos) in gray. Box plots show min, 25^th^ percentile, median, 75^th^ percentile and max. Scatterplots show individual data points (dots) with median and inner quartiles (line and error bars). *p < 0.05, **p < 0.01, ***p < 0.001.

To silence the vmPFC in males, we infused an adeno-associated virus (AAV) containing the gene for an inhibitory Gi-coupled hM4Di receptor into the vmPFC (prelimbic and infralimibic areas) to express DREADD receptors (Fig. 2a,b). DREADD-selective, biologically inert ligand, clozapine-N-oxide (CNO) was injected i.p. prior to testing, which selectively silenced the infected neurons. Rats in the 3 groups: DREADD + CNO, DREADD + Vehicle, Control Virus (AAV with EGFP-only) + CNO (Fig. 2c) acquired and extinguished CRs to the tone similarly (Supplementary Fig. 2). On tests, the DREADD + CNO group failed to show renewal responding with similar low responding to CSs in both contexts, while DREADD + Vehicle and Control Virus + CNO groups showed renewal responding with higher induction of CRs to the CSs in the acquisition context (Fig. 2d–f). A planned orthogonal contrast of the elevation difference (t(15) = 2.248, p = 0.040) confirmed that silencing vmPFC neurons disrupted renewal behavior in males (Fig. 2f). Responding during preCS remained low and did not differ between groups (*p* > 0.05).

In a subset of rats, we examined Fos induction during a test for renewal in the acquisition context to verify differential activation in the DREADD + CNO group compared to the control groups (Fig. 2g–j). The AAV expression was similar across groups (Fig. 2h). The percentage of AAV neurons that were double-labeled with Fos (AAV + Fos/AAV) was lowest in the DREADD + CNO group; however, the decrease was not significantly different from the controls (mean ± SEM by group: DREADD + CNO: 35 ± 5%, DREADD + vehicle: 42 ± 6%, Control Virus + CNO: 38 ± 3%; *p* > 0.05). Interestingly, the DREADD + CNO group had significantly higher number of total Fos positive neurons compared to controls (Fig. 2i; planned orthogonal contrast, *t*(8) = −3.429, *p* = 0.009), possibly due to disinhibition of neurons receiving inhibitory inputs from the hM4Di infected neurons. Furthermore, the percentage of total Fos expressing neurons that were double-labeled (AAV + Fos/Fos) was significantly lower in the DREADD + CNO group compared to controls (Fig. 2j; planned orthogonal contrast, *t*(8) = 5.616, *p* = 0.001), further suggesting increased total Fos may be due to a release from inhibition by hM4Di infected neurons.

### Experiment 3

**Figure 3:**
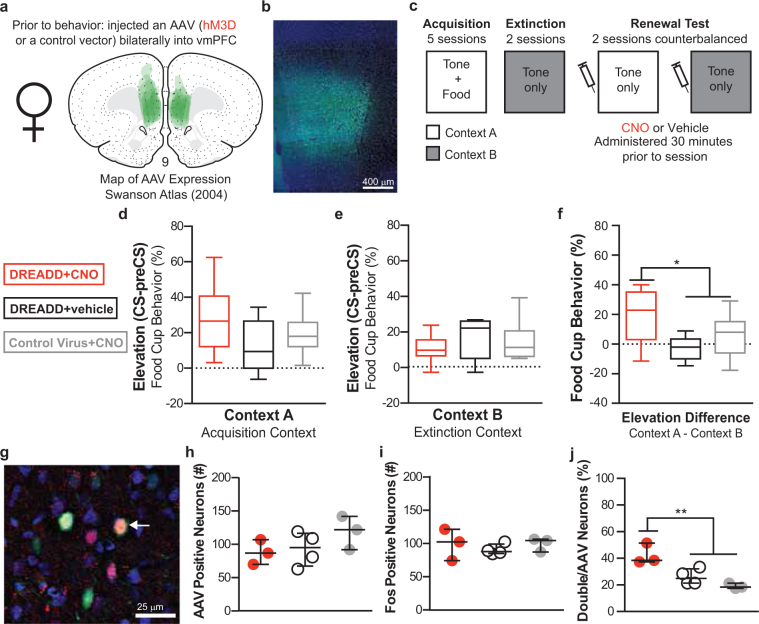
Chemogenetic vmPFC stimulation in female rats during renewal of conditioned responding to a food cue. The extent of AAV expression within the vmPFC is shown on a modified atlas template (Swanson 2004) (**a**) and a representative image (**b**). Experimental design (**c**). Conditioned responding in the acquisition context (**d**) and the extinction context (**e**) and as a difference in responding between the two contexts (**f**). Representative image of AAV-positive (green), Fos-positive (magenta), and double-labeled (arrow) neurons (**g**). Total number of AAV-positive (**h**) or Fos-positive (**i**) neurons. Percentage of AAV-positive neurons that were double labeled (**j**). DREADD + CNO group (n = 8 behavior, n = 3 Fos) in red, DREADD + vehicle (n = 5 behavior, n = 4 Fos) in black, Control Virus + CNO (n = 6 behavior, n = 3 Fos) in gray. Box plots show min, 25^th^ percentile, median, 75^th^ percentile and max. Scatterplots show individual data points (dots) with median and inner quartiles (line and error bars). *p < 0.05, **p < 0.01.

To activate vmPFC neurons in females, we infected vmPFC neurons with AAV containing hM3Dq vector and then trained the rats in the same behavioral design as described above (Fig. 3a,b; Experiment 3). All rats acquired and extinguished CRs similarly (Supplementary Fig. 3). At test, the DREADD + CNO group had higher CRs to the CSs in the acquisition context (Fig. 3d–f; planned orthogonal contrast of the elevation difference t(16) = −2.528, p = 0.022.), while control groups had similar responding to the CSs in both contexts. Responding during preCS remained low and did not differ between groups (*p* > 0.05). Stimulating vmPFC neurons in females induced renewal of behavior, thereby eliminating behavioral sex differences. Similar to Experiment 2, to confirm hM3Dq effectiveness, we analyzed Fos induction during a test for renewal in the acquisition context. We found no differences in the total number of AAV labeled or total Fos labeled neurons (*p* > 0.05). In the DREADDs + CNO group compared to controls there were significantly more double-labeled neurons (AAV + Fos) (planned orthogonal contrast, *t*(7) = −3.649, *p* = 0.008, data not shown), and a significantly higher percentage of total AAV-labeled neurons were double labeled with Fos (AAV + Fos/AAV) (Fig. 3j; planned orthogonal contrast, *t*(7) = −4.787, *p* = 0.002). In addition, a higher percentage of total Fos labeled neurons were double labeled (AAV + Fos/Fos) in the DREADDs + CNO group compared to controls (mean ± SEM by group: DREADD + CNO: 39 ± 8%, DREADD + vehicle: 25 ± 4%, Control Virus + CNO: 23 ± 6%; planned orthogonal contrast, *t*(7) = −2.526, *p* = 0.039).

## Discussion

These findings are the first evidence that manipulating vmPFC activity can impact behavioral sex differences. Chemogenetic silencing of vmPFC neurons in male rats disrupted context-mediated renewal of cue driven food seeking while stimulating vmPFC neurons in female rats induced renewal of this behavior, demonstrating vmPFC activity is crucial for renewal in both sexes. These results reveal a likely sex-specific function of the vmPFC in context-mediated renewal, a model of persistent food seeking relevant to environmentally driven appetites. Nevertheless, whether manipulations in the opposite direction (i.e., activating the vmPFC in males and inhibiting it in females) would have an effect on context-mediated renewal of responding to food cues remains to be tested.

The current evidence of vmPFC’s function during renewal of responding to food cues is in agreement with prior evidence for the vmPFC’s role in the control of appetitive behaviors by associative cues in males. The vmPFC is recruited during cue-food associative learning^29^, is critical for context-mediated renewal of instrumental responding for drug and food^30–32^, and feeding stimulated by contextual food cues^33,34^. However, the vmPFC is not solely important for contextual processing, it is also recruited by discrete cues during learning and cue-potentiated feeding^35,36^. It seems likely the vmPFC function involves accessing different memory traces in order to guide feeding behavior.

Here, during extinction, the levels of conditioned responding were somewhat low initially compared to responding during acquisition and therefore instead of a traditional extinction curve there was overall low responding. Previously, in a between-subjects ABA preparation, we found rats tested in a novel context during extinction had lower conditioned responding compared to rats tested in the acquisition context^19^. In the current study all rats were placed in a novel context during extinction training, which is likely the reason for their low responding.

The lack of renewal in females may be due to differences in learning strategies or differences in the brain mechanisms mediating contextual learning and extinction. Females may be relying more on the extinguished cue-food association than contextual information and thus generalizing across contexts more than males. In support of the idea that differences in extinction learning or recall may be mediating renewal differences, sex differences have been found during fear extinction learning^37,38^. In general, females showed better fear extinction learning; however, other evidence suggests the differences are more complex^22,39,40^. Furthermore, in a preparation that tested food consumption in the presence of a fear cue (a tone predictive of electric footshocks), during extinction testing, female rats showed longer inhibition of feeding compared to males^41^. Finally, a study that compared freezing behavior and food consumption in neutral and aversive contexts also found sex differences in context generalization^23^. Males and females showed similar freezing behavior (high in aversive and low in neutral contexts), indicating similar context discrimination across sexes, but only males selectively inhibited feeding in an aversive context, while females inhibited feeding in both contexts.

Our prior work^19,20^ demonstrated that renewal of responding in females is modulated by estradiol. Estradiol replacement to ovariectomized rats induced renewal responding compared to intact and ovariectomized females without estradiol replacement. Interestingly, testing rats during different estrous phases did not impact renewal responding in females, indicating that estradiol effects are not mediated acutely during renewal expression. Instead, estradiol may be important during extinction learning, similar to its impact on fear extinction^37^. In that regard, previous fear conditioning work found that higher generalization across contexts in females is in part dependent on estradiol^42^.

The vmPFC’s function in renewal may be mediated via glutamatergic pyramidal neurons or inhibitory interneurons or both^43,44^. Recent work has suggested that there is heterogeneity in the vmPFC substrates controlling different aspects of feeding behavior, and that AMPA- but not NMDA-type glutamatergic receptors are critical for behavioral organization during feeding^45^, while μ opioid system mediates food consumption, likely via changes in motivation and palatability^46^. Interestingly, μ opioid receptors are located on GABA neurons and it was hypothesized that their stimulation disinhibits the pyramidal output^46^ indicating that at least some aspects of feeding behavior are mediated by GABA transmission. Similarly, orexin receptor 1 blockade during cue-potentiated feeding resulted in increased Fos induction in the vmPFC^36^ also indicating inhibitory mechanisms. Finally, GABA transmission in the medial prefrontal cortex increased when male rats were placed in a cocaine-associated environment^47^. Therefore, inhibitory mechanisms may be critical during renewal responding, which would explain the unexpected Fos induction patterns we found using inhibitory DREADDs in male rats (Experiment 2). Inhibition of inhibitory neurons could have caused disinhibition of their targets and higher Fos induction in the experimental (hM4Di) group. However, future work is needed to determine if hM4Di inhibition of the vmPFC during renewal silenced GABA neurons and to determine which glutamatergic inputs were interrupted. In that regard, our recent study found the vmPFC inputs from the ventral hippocampal formation, paraventricular nucleus of the thalamus and basolateral amygdala were differently recruited during context-mediated renewal in males and females, suggesting under-recruitment in females may be the reason for lack of renewal (Anderson, L. C. & Petrovich, G. D., *Neurobiology of learning and memory*, Under Review (2018)).

These novel results are expected to propel future molecular and translational work investigating sex differences in associative learning and memory and how learned cues contribute to maladaptive eating habits and eating disorders. This work points to the vmPFC as a novel neural target for therapeutic intervention and sex-specific treatment relevant to sex differences in obesity and maladaptive eating behaviors^16^. More generally, this work suggests dissociable vmPFC functioning in males and females in decision-making processes that guide our behavior^48,49^.

## Methods

### Subjects

Adult male and female Long-Evans rats (250–275 g at arrival; total n = 88; Charles River Laboratories) were individually housed and maintained on a 12 h light/dark cycle (lights on at 7:00). Males and females were housed in separate colony rooms. 16 rats (8 males, 8 females) were used for Experiment 1. 36 males were used for Experiment 2; 36 females were used for Experiment 3. 35 rats total were excluded for the following reasons: poor health (1 male from Experiment 2, 1 female from Experiment 3), misplaced/insufficient viral expression (17 males from Experiment 2, 15 females from Experiment 3), or high preCS responding (1 female from Experiment 3; preCS responding higher than 3 standard deviations from the mean). Subjects in Exp. 2 and 3 underwent surgery after they were acclimated to the colony room for two days. Animals were given a week to recover post-surgery during which they were weighed and handled daily. All rats (Exp. 1–3) were given *ad libitum* access to standard laboratory chow (18% Protein Rodent Diet #2018, Harlan Teklad Global Diets, Madison, WI) and water, except as otherwise noted. All housing and testing procedures were in compliance with the National Institute of Health Guidelines for Care and Use of Laboratory Animals and were approved by the Boston College Institutional Animal Care and Use Committee. None of the animals were used in prior experimental procedures unrelated to these studies. Animals were randomly assigned to experimental condition and renewal test day order (see Behavioral Training Procedure).

### Surgical Procedure

Viral stocks of adeno-associated virus (AAV) carrying constructs AAV5-hSyn-HA-hM4D-IRES-mCitrine (Experiment 2), AAV5-hSyn-HA-hM3D-IRES-mCitrine (Experiment 3), and AAV5-hSyn-EGFP (Experiment 2 and 3) were purchased from the Vector Core at University of North Carolina, Chapel Hill. Animals were briefly anesthetized with isoflurane (5%; Baxter Healthcare Corporation, Deerfield, IL, USA), then deeply anesthetized with an intramuscular injection of a mixture (1 ml/kg body weight) of ketamine (50 mg/mL; Fort Dodge Animal Health, Fort Dodge, Iowa) and xylazine (10 mg/mL; LLOYD Laboratories, Shenandoah, IA, USA). While under anesthesia, animals received bilateral stereotaxically placed injections of the AAVs into the ventromedial prefrontal cortex (vmPFC; volume 0.75 µl; flow rate 0.25 µl/min; titer 1 × 10e12, relative to bregma anterior-posterior [AP]: +2.8 mm, mediolateral [ML]: +/−0.7 mm, dorsoventral [DV]: −4.7 mm). A 10 µl Hamilton syringe with 32 gauge cannula driven by a motorized stereotaxic injector (Stoelting, Wood Dale, IL) was used to deliver microinjections. Stereotaxic surgeries were performed according to the procedures for aseptic technique in survival surgery and postoperative care approved by Boston College IACUC. The Biosafety Committee at Boston College has approved all protocols with AAVs. Behavior started three weeks after surgery to allow for recovery and sufficient expression of the viruses.

### Apparatus

The behavioral training was conducted in identical behavioral chambers (30 × 28 × 30 cm; Coulbourn Instruments, Allentown, PA) located in a room different from the colony housing rooms. The chambers had aluminum top and sides, clear Plexiglas rear wall and front hinged door and a floor of stainless steel rods 5 mm thick spaced 15 mm apart. Chambers contained a recessed food cup (3.2 × 4.2 cm) and a 4 W house light. Each chamber was located in a sound- and light-attenuating cubicle (79 × 53 × 53 cm), which was equipped with a ventilation fan (55 dB) and video camera attached to a recording system (Coulbourn Instruments, Allentown, PA). The conditioned stimulus (CS) was a 10 second tone (75 dB, 2 kHz). The unconditioned stimulus (US) consisted of two food pellets (45 mg pellets, formula 5TUL; Test Diets, Richmond, IN, USA) delivered to the food cup. Chambers were modified in visual, tactile, and olfactory features, to create two distinct environments. For Context 1, a black Plexiglas panel was placed on top of the grid floor (so that rats could not see or feel the grids), and the doors to the cubicles were closed. For Context 2, a black Plexiglas panel was inserted diagonally across the side of the chamber creating a wall, and the doors to the cubicle were left open, and 1% acetic acid in water solution (Fisher Scientific, Fair Lawn, NJ) was sprayed onto the tray below the grid floor.

### Behavioral training procedure

All behavioral training and testing occurred between 9:00 and 14:00. A week before start of training, all rats were food deprived and their daily food allotment was restricted to gradually reach 85% of their body weight; they were maintained at this weight for the duration of the experiment. All rats received 1 g of the food pellets (Unconditioned Stimulus, US) in the home cage the day before the training started to familiarize them with the pellets. The training consisted of three phases: conditioning (acquisition), extinction, and renewal tests. The conditioning and extinction occurred in two different contexts and the testing was conducted in both contexts (test order counterbalanced). The behavioral chambers used as distinct contexts for conditioning and extinction were counterbalanced (the acquisition context for half of the rats was in Context 1 and for the other half was in Context 2). During the acquisition phase, rats were trained for five days, with one 34-minute training session per day. During each session they received eight presentations of the tone CS, each immediately followed with delivery of food pellets (US) into the food cup. During the extinction, rats received two 34-minute sessions (one session per day), each with eight presentations of the tone CS alone with no USs. Rats were tested for renewal of responding with CS-only presentations in Context A and Context B, counterbalanced for order, on separate days (order randomly assigned). The tests for renewal were 34-minute sessions with eight CS presentations with no USs. All sessions were recorded and stored on DVDs for behavioral analysis. In Experiments 2 and 3, prior to the tests for renewal, clozapine-N-oxide (CNO; Enzo Life Sciences, Farmingdale, NY) was injected i.p. in half of the animals who received AAV5-hSyn-HA-hM4D-IRES-mCitrine (Experiment 2) or AAV5-hSyn-HA-hM3D-IRES-mCitrine (Experiment 3), the other half received saline. All of the animals that received the control viral vector (AAV5-hSyn-EGFP) received CNO. This resulted in 3 groups: DREADDs + CNO, DREADDs + Vehicle, Control Virus + CNO. CNO was administered at a dose of 3 mg/kg (1 mg/ml).

### Behavioral Measures

Trained observers, ‘blind’ to experimental condition or sex of the rats, analyzed animals’ behavior from the video recordings. The primary measure of conditioning (conditioned response, CR) was the expression of ‘food cup behavior’ during the CS. The food cup behavior was defined by distinct nose pokes into the recessed food cup, or by rats standing in front of and directly facing the food cup. Behavior was scored every 1.25 seconds during each 10 second preCS and CS periods. At each observation only one behavior was recorded (food cup or other). The number of CRs was summed and converted to a percentage of the total time during each period an animal expressed food cup behavior.

### Histological Procedures

After the end of renewal tests in Exp. 2 and 3, rats were anaesthetized with tribromoethanol (375 mg/kg body weigh, intraperitoneal injection) and transcardially perfused with 0.9% saline followed by 4% paraformaldehyde in 0.1 M borate buffer. A subset of rats (n = 4 per group) was perfused ninety minutes after the end of renewal test to be used for Fos induction detection. The brains were stored for 20–24 hrs at 4 °C in a paraformaldehyde and 12% sucrose mixture and then rapidly frozen in hexanes cooled with dry ice and stored at −80 °C. Brains were cut into 40 μm coronal sections using a microtome and collected into three adjacent series. For all brains, one tissue series was mounted unstained for identification of viral expression in the vmPFC. Another series was mounted and stained with thionin for identification of cytoarchitectonic borders. For the brains used for Fos detection, one tissue series was immediately processed with Fos immunohistochemistry, described below.

### Tissue Processing and Immunohistochemistry

Immediately following slicing, sections were incubated for 72 h at 4 C in a blocking solution [KPBS containing 0.3% Triton X-100, 2% normal donkey serum (017-000-121; Jackson ImmunoResearch, West Grove, PA, USA)], with the primary antibody: c-Fos goat primary (1:2,000; sc-52-G; Santa Cruz, Dallas, TX). After rinses in KPBS, tissue was incubated for 1 h in the dark in the blocking solution containing the secondary antibody: Alexa 546 anti-goat (1:200; A11056; Invitrogen, Carlsbad, CA, USA). Following rinses in KPBS, in semidarkness tissue was mounted onto slides (SuperFrost Plus), dried, coverslipped with Vectashield HardSet Mounting Medium with DAPI (H-1500; Vector Labs, Burlingame, CA, USA), and stored at 4 C until analysis. This immunohistochemistry procedure was successfully repeated 8 times in the current study, and has been used successfully numerous additional times within our laboratory.

### Image acquisition and analysis

The acquisition of images was conducted with a Zeiss Axio Image Z2 fluorescence microscope (Carl Zeiss Microscopy GmbH; Jena, Germany) and attached Hamamatsu ORCA-R2 camera (Bridgewater, NJ, USA). To determine the location and extent of the AAV expression, images of the areas with viral expression and the adjacent thionin-stained tissue were acquired at 10x. Neuroanatomical borders were drawn onto the thionin-stained image and then transposed to the adjacent fluorescent image using ImageJ software (NIH). The spread of AAV expression were then drawn on computerized versions of the standard rat brain atlas templates^50^ using illustration software (Adobe Illustrator CS5.5). Based on this analysis, well-defined and localized AAVs expressions were identified and included for data analysis (at least 50% in the prelimbic and infralimbic regions at bregma AP +3.60, +3.20 or +2.80; with less than 25% of the injection spread outside this target region). Only these subjects were included in behavioral data analysis. For Fos analysis, 2 × 2 tiled images were taken at 20× (tissue area approximately 820 × 630 μm) in the prelimbic area of the vmPFC (+3.20 mm AP from bregma). Three brains were excluded from Fos analysis (one male Control Virus + CNO from Experiment 2, one female DREADD + CNO and one female Control Virus + CNO from Experiment 3) due to inadequate viral expression in the prelimbic area at bregma +3.20 mm AP. Images were pseudocolored with green for DREADDs, red for Fos and blue for DAPI (nuclear counterstain). Single AAV, single Fos and double-labeled (AAV and Fos) cells were determined based on characteristic nuclear (Fos) or cytoplasmic (AAV) labeling by an investigator ‘blind’ to experimental condition. Single (AAV or Fos) and double-labeled (AAV and Fos) cells were counted. Double-labeled cells were expressed as a percentage of total AAV cells and total Fos cells.

### Statistical analysis

Behavioral data analysis was conducted to compare CRs using a mixed-design ANOVA (repeated (context) and between (sex)) and significant main effects and interaction effects (*p* < 0.05) were followed by *post hoc* two-tailed t-tests. In addition, in Experiments 2 and 3, *a priori* planned orthogonal contrasts were used. All statistical analyses were conducted in SPSS. Data are presented as median and inner quartiles, as well as minimum and maximum when indicated. Statistical details of the experiments can be found in the figure legends and figures. Exclusion of subjects was determined based on the criteria described under Subjects and Image acquisition and analysis. Sample sizes were determined based on previous research in our lab^19,20^. All relevant data from the current study are available from the corresponding author upon request.

## Electronic supplementary material

Supplementary Figures
