# Supplementary material for: Ventromedial prefrontal cortex mediates sex differences in persistent cognitive drive for food
Source: Sci Rep. 2018 Feb 2;8:2230. doi: 10.1038/s41598-018-20553-4 (PMC5797070; doi:10.1038/s41598-018-20553-4)
Supplement: Supplementary file 1 — Supplementary Figures [file 41598_2018_20553_MOESM1_ESM.pdf]

Supplementary Figures

Ventromedial prefrontal cortex mediates sex differences in persistent cognitive drive for food

Anderson, L.C. & Petrovich, G.D.

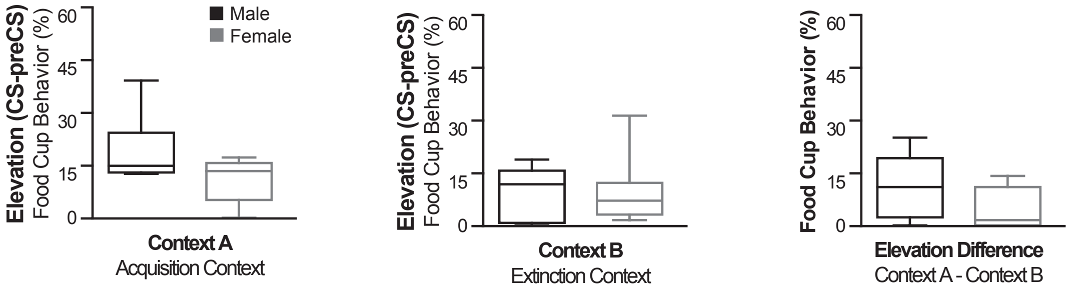

Supplementary Figure 1. Conditioned responding in male and female rats during renewal tests in Experiment 1. Elevation scores represent responding (food cup behavior) during CS minus preCS in the Acquisition Context, Extinction Context and the Elevation Difference (Elevation score in the Acquisition context minus Elevation score in the Extinction context). There were no differences between the sexes ($p > 0.05$). n=8 for both males (shown in black) and females (shown in gray). Box plots show min, 25th percentile, median, 75th percentile and max.

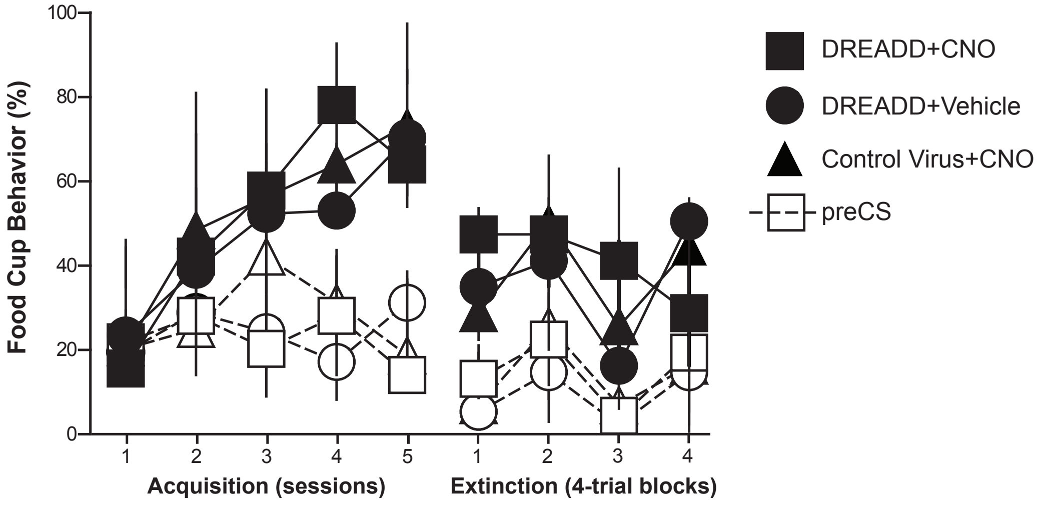

Supplementary Figure 2. Conditioned responses during acquisition and extinction in Experiment 2. Percentage of time male rats expressed food cup behavior (median, error bars=inner quartiles) during the preCS (open) and CS (filled) periods across training sessions. Acquisition is shown as the average responding during each session. Extinction is shown as the average responding in 4-trial blocks (2 blocks per session; blocks 1&2 were trials during Session 1 and blocks 3&4 during Session 2). Acquisition and extinction training occurred in different contexts. DREADD+CNO group (n=7) represented with squares, DREADD+vehicle (n=6) represented with circles, Control Virus+CNO (n=5) represented with triangles. There were no group differences during Acquisition or Extinction (p>0.05).

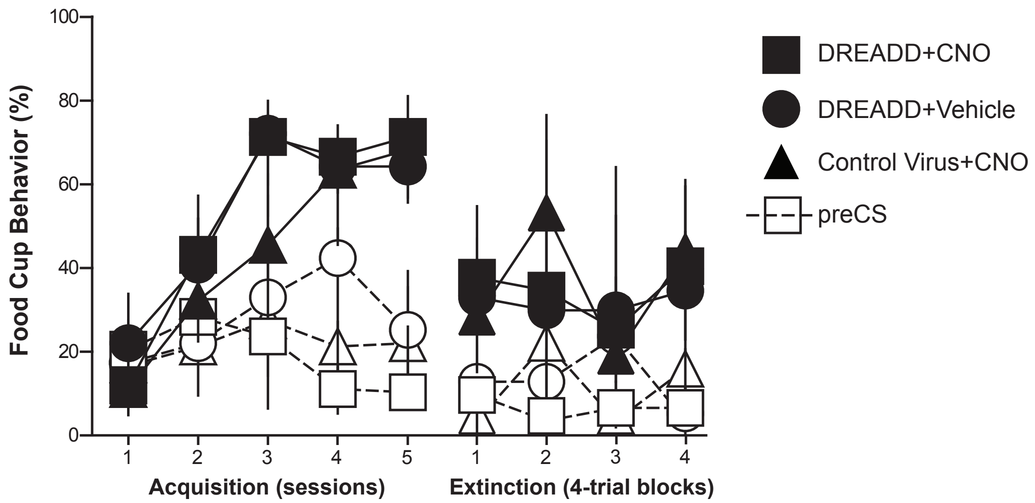

Supplementary Figure 3. Conditioned responses during acquisition and extinction in Experiment 3. Percentage of time female rats expressed food cup behavior (median, error bars=inner quartiles) during the preCS (open) and CS (filled) periods across training sessions. Acquisition is shown as the average responding during each session. Extinction is shown as the average responding in 4-trial blocks (2 blocks per session; blocks 1&2 were trials during Session 1 and blocks 3&4 during Session 2). Acquisition and extinction training occurred in different contexts. DREADD+CNO group (n=8) represented with squares, DREADD+vehicle (n=5) represented with circles, Control Virus+CNO (n=6) represented with triangles. There were no group differences during Acquisition or Extinction ($p>0.05$).
